# Measuring 3D Video Quality of Experience (QoE) Using A Hybrid Metric Based on Spatial Resolution and Depth Cues

**DOI:** 10.3390/jimaging9120281

**Published:** 2023-12-18

**Authors:** Sahin Coskun, Gokce Nur Yilmaz, Federica Battisti, Musaed Alhussein, Saiful Islam

**Affiliations:** 1 Department of Electrical-Electronics Engineering, Graduate School of Natural and Applied Sciences, Gazi University, Ankara 06560, Turkey; sahin.coskun@gazi.edu.tr; 2 Department of Computer Engineering, TED University, Ankara 06420, Turkey; 3 Department of Information Engineering, University of Padova, 35131 Padova, Italy; 4 Department of Computer Engineering, College of Computer and Information Sciences, King Saud University, P.O. Box 51178, Riyadh 11543, Saudi Arabia

**Keywords:** 3D video, stereoscopic vision, human vision system, quality of experience, 3D-video QoE evaluation metric, numerical methods

## Abstract

A three-dimensional (3D) video is a special video representation with an artificial stereoscopic vision effect that increases the depth perception of the viewers. The quality of a 3D video is generally measured based on the similarity to stereoscopic vision obtained with the human vision system (HVS). The reason for the usage of these high-cost and time-consuming subjective tests is due to the lack of an objective video Quality of Experience (QoE) evaluation method that models the HVS. In this paper, we propose a hybrid 3D-video QoE evaluation method based on spatial resolution associated with depth cues (i.e., motion information, blurriness, retinal-image size, and convergence). The proposed method successfully models the HVS by considering the 3D video parameters that directly affect depth perception, which is the most important element of stereoscopic vision. Experimental results show that the measurement of the 3D-video QoE by the proposed hybrid method outperforms the widely used existing methods. It is also found that the proposed method has a high correlation with the HVS. Consequently, the results suggest that the proposed hybrid method can be conveniently utilized for the 3D-video QoE evaluation, especially in real-time applications.

## 1. Introduction

The Quality of Experience (QoE) of a video is a measure that states the satisfaction level from a viewer’s perspective. Hence, this measurement is viewer-centric and focuses on measuring the overall satisfaction and acceptability of a video by taking a holistic approach by evaluating all QoE factors that can affect a viewer’s appreciation positively and/or negatively [1].

The QoE is based on real and end-user experiences. Therefore, the QoE is directly affected by objective and subjective parameters. The objective parameters are the parameters that originate from Quality of Service (QoS) factors and depend mostly on network performance, software, and hardware features. On the contrary, the subjective parameters are determined by the influence of the viewers` individual preferences, expectations, previous video experiences, etc. So, the subjective parameters are more difficult to categorize compared to the objective parameters. However, they are more likely to arise from different perception characteristics that people have (e.g., age, eyesight, mobility, perspective, etc.). For this reason, it is indisputable that the measurement of the subjective parameters is more arduous because they are more abstract. In addition, the other challenge is the design of a comprehensive QoE metric. To be able to design a comprehensive QoE metric, a sufficient number of QoE factors is required. These factors are possibly controlled, measured, or simply collected and reported [2].

The video quality perceived by a viewer is considered the most important part of the QoE [3]. Three-dimensional videos are special types of video representations that can enable a feeling of being in the same space while viewing them due to the addition of depth perception with the depth cues being forward. It is clear that in addition to the quality, a vital factor affecting the QoE in 3D videos is the perception of depth enabled by the viewer. Therefore, the key to increasing the QoE of 3D videos for a viewer is to enable 3D video representations that will create a plausible depth perception in the viewer. 

As is known, depth perception is the main result of the stereoscopic vision process carried out by the HVS [4,5]. Except for people with various visual impairments or losses, every person with normal vision has an HVS that combines monocular and binocular depth cues to achieve stereoscopic vision. Although this system works according to the same principles in every human, the perceived depth may be relativistic because of different perception characteristics and visual experiences. In other words, it is possible to have different QoE evaluations about a 3D video when viewed by different viewers due to different credibility perceptions enabled. This is a major obstacle to performing an accurate QoE evaluation for the 3D videos.

Currently, the QoE evaluation for 3D videos can be performed by using two methods [5]. One of them relies on the subjective evaluation in which real human observers assess the 3D-video QoE. It is a fact that the subjective quality evaluation is vital for accurately assessing the 3D-video QoE. However, the subjective evaluation is difficult to perform due to being time consuming and costly and its unsuitability for real-time applications [6,7]. The other one relies on the objective QoE in which iterative mathematical and statistical metrics are utilized during the evaluation process. The objectivity of these metrics stems from their rational expansions that are accepted by the researchers and enable reliable and objective evaluations on a regular basis. These metrics mostly do not consider the most important characteristics of the HVS for 3D-video perception. Therefore, they generally do not achieve a high correlation with real-human quality evaluations [8].

The subjective and the objective image or video QoE metrics existing in the literature can be categorized as full reference (FR), reduced reference (RR), and no reference (NR) [9,10,11,12]. The FR ones cannot be used without the original video, and the RR ones require video features obtained from the original video. Therefore, it is not possible to run the FR and the RR metrics simultaneously with a streaming video. On the other hand, the NR metrics do not require an original video or video features obtained from the original video for the QoE evaluation. It means that they can run simultaneously with a streaming video. However, the FR and the RR metrics can contain more information for the QoE evaluation than the NR metrics. Therefore, the FR, the RR, and the NR metrics have superiorities over each other in terms of the QoE evaluation. Another problem with the subject is that researchers feel obliged to select one of these three approaches when developing metrics. Also, it has been observed that pseudo reference image (PRI) quality-evaluation metrics have been developed in recent years. Contrary to conventional FR, RR, and NR metrics, the PRI metrics use a new type of reference. In conventional metrics, the reference is the original image, which is assumed to have a perfect quality or some derived characteristics of the original image. However, in the PRI metrics, the reference, which is called the pseudo reference image, is generated from the distorted image by further degrading it in several ways and to certain degrees [13,14]. With this approach, the PRI metrics have brought a new breath to the image-quality-evaluation field.

On the other hand, image-quality-evaluation approaches need to be developed according to the characteristics of the digital images obtained by different rendering methods. In general, it is possible to classify digital images into three types according to rendering methods: Natural Scene Image (NSI), Computer Graphic Image (CGI), and Screen Content Image (SCI). The NSIs are digital images captured from the real world and may be degraded by physical reasons such as a low-quality lens, being out-of-focus, motion blur, and insufficient and inappropriate lighting conditions and aerial conditions. CGIs are created or animated by using computer software and are widely used in video games, animations, simulators, etc. They may be degraded by rendering artifacts. SCIs are composite images and consist of texts, graphics, icons, etc. Also, they sometimes contain NSI and CGI regions so that they may be degraded by NSI and CGI degraders. Computer-generated SCIs and CGIs have more noise-free smooth areas, high-saturation color content, repeated patterns, and low- or high-frequency contents [15,16]. As can be seen, it is significant to measure the quality of images that are likely to be dominated by different defects due to differences in the rendering methods by using the video-quality-evaluation method specific to the image’s type. Otherwise, the quality measurements may be inaccurate.

There are also metrics developed to deal with some physical drawbacks that reduce the visual quality and the viewer’s depth perception. One of them is the metric that is developed for measuring the light field. What exactly we can see depends on our precise position in the light field. The light field records the total of all light rays in 3D space that flow through every point and in every direction. Therefore, the light field contains very rich information. A light-field image contains many depth cues to make depth estimation possible. The light-field quality metrics measure the light-field qualities of the light-field images [17,18,19,20].

Additionally, in order to increase the viewer’s depth perception, developing objective quality-evaluation metrics for dehazed images has been a leading light recently. There are many image-dehazing algorithms to remove the haze from the images captured in hazy conditions and preserve the intrinsic image structures. To assess and compare the image-dehazing algorithms, subjective and objective methods can be used. Since subjective evaluation is a time-consuming process and difficult to apply, objective quality-evaluation metrics are more preferable for the researchers [21,22].

Audio–visual content-quality-evaluation issues have also been researched for decades because visual signals are rarely presented without accompanying audio. The distortions that may separately (or conjointly) afflict the visual and audio signals collectively shape the user-perceived Quality of Experience (QoE) [23,24,25].

Lastly, with the recent rapid developments in the field of virtual reality, developing a 360-degree image (also known as an omnidirectional, panoramic, or virtual-reality image) quality-evaluation metric has been a remarkable research area. Three hundred and sixty-degree images and videos include visual information covering the entire 180 × 360° viewing spherical. Hence, compared to conventional 2D spaces, there are many challenges to developing a quality metric for immersive multimedia. Especially, ultrahigh or even higher resolution requirements and degradations in 360-degree images/videos are the main two challenges. In the quality-evaluation field of 360-degree images and videos, multichannel convolutional neural networks (CNNs) have been successfully used due to their good performance [26,27,28]. 

In light of the above explanations, it can be clearly comprehended that the objective QoE metrics, which are frequently used today, are not adequate for the 3D-video QoE evaluation. Hence, there is a need to develop a 3D-video QoE evaluation metric that has a high correlation with the HVS. While developing this metric, a QoE-based approach that examines with the effects of real visual experiences and different perception characteristics of humans on depth perception should be utilized. On the other hand, designing a hybrid 3D-video QoE evaluation combining the superiorities of the FR, the RR, and the NR metrics is a remarkable advantage. The development of a 3D-video QoE evaluation metric with all these properties contributes to the production of more scientific studies on ubiquitous 3D-video technologies.

Considering all of these facts, a hybrid 3D-video QoE evaluation metric relying on the depth cues associated with the spatial-resolution feature of a 3D video, which is quite effective at influencing the depth-perception experience of a 3D viewer, is proposed in this study. These depth cues are determined as the blurriness and motion information extracted from the 2D-texture videos and retinal-image size and convergence extracted from the depth maps (DMs). As the first step of the proposed-metric-development process, prediction models are developed for these depth cues. Due to the nonobjective features of the 3D videos, such as the perceived depth and naturalness, which differ from person to person, subjective tests are applied to evaluate the QoE of the 3D videos. Then, the depth cues and the Mean Absolute Score (MOS) values obtained from the subjective tests are subjected to a correlation analysis to form the proposed hybrid 3D-video QoE evaluation metric. The performance-evaluation results derived by using the proposed metric prove its effectiveness in assessing the 3D-video QoE.

The rest of this paper is organized as follows: Section 2 includes state-of-the-art studies. Section 3 explains the proposed hybrid 3D-video QoE evaluation metric. Section 4 includes the results and the discussions. This paper is ended with the conclusions and future works given in Section 5. 

## 2. State-of-the-Art Studies

In this section, we provide an overview of the existing studies in the literature in two parts, adhering to the reference-classification approach in Section 1. In the first part, the FR and the RR metrics are presented together, which need to take the original video or some features of the original video as a reference, respectively. The second part includes the NR metrics, which do not need any references for the video-quality-measurement process. Finally, we present an evaluation of the state-of-the-art studies to identify the literature gap. 

### 2.1. Reference-Based Metrics

In [29], the use of objective two-dimensional (2D) video-quality metrics for the 3D-video-quality assessment (VQA) is discussed, and a perceptual-based objective metric that mimics the HVS is proposed. In this study, the luminance component is taken as an input parameter in the development of the metric. According to the experimental results, it is found that using 2D- and 3D-video-quality evaluations is appropriate since the proposed Perceptual Quality Metric (PQM) mimics the MOS and has greater alignment with it compared to the Video Quality Metric (VQM). To the FR metric in [30], the HVS properties, such as the contrast-sensitive function and luminance masking, are taken into account, and in order to analyze the perceptual similarity of the blocks in the left and right views of the stereoscopic video frames, 3D-DCT transform is used. In [31], a 3D structural-similarity (3D-SSIM) approach is proposed. The proposed algorithm regards a video signal as a 3D volume image and combines a local SSIM-based quality measure with local information content and distortion-based pooling methods. The proposed metric in [32] uses blocking artifacts, blurring in edge regions, and the video-quality difference between two views. The proposed metric in [33] uses the color-video information and the depth information as the input parameters. The color-quality metric (CQM) for 3D videos proposed in [34] takes the luminance coefficient into consideration as it is much more sensitive than the chrominance coefficient of a frame for the HVS. In [35], the proposed metric focuses on the interview correlation of the spatial–temporal structural information extracted from adjacent frames. In [36,37], the proposed FR 3D-video-quality metric is modeled around the HVS, fusing the information of both the left and right channels and considering color components, the cyclopean views of the two videos, and the disparity. Since the metric also considers the screen size, video resolution, and the distance of the viewer from the screen, it is possible to use this metric in different applications. In [38,39], an RR stereoscopic VQA metric is proposed, which comprises spatial neighboring information from the contrast of grey-level co-occurrence matrices for both color and depth and edge properties. 

In [40], an FR stereoscopic video-quality-assessment (SVQA) metric based on the Stereo Just-Noticeable Difference (SJND) model that works by using contrast, spatial masking, temporal masking, and binocular masking factors to mimic the HVS is proposed. In [41], an FR stereoscopic VQA metric is proposed by using measurements of structural distortions, blurring artifacts, and content complexity. In the FR metric proposed in [42], human stereoscopic vision is modeled by combining left-eye-view and right-eye-view information through 3D-DCT transformation, and the contrast sensitivity of the HVS is considered as well as the depth information of the scene. The metric proposed in [43] is developed by incorporating the stereoscopic visual-attention (SVA) metric into the stereoscopic video-quality-assessment (SVQA) metric in order to benefit the image-quality-evaluation metrics. The proposed metric in [44], in which the SSIM metric is adapted to stereoscopic videos, is the product of approaches that combine SSIM maps and depth maps with local and global weighting methods. In [45], with the approach that the 3D distortions affecting the 3D video quality should also be taken into account when developing a 3D VQA metric, the proposed metric uses texture distortions (i.e., ghost effects and contour artifacts) and depth distortions as the input parameters. In [46], an FR 3D VQA metric based on the dependencies between motion and its binocular disparities was developed. This metric calculates the spatial, temporal, and depth features and uses them in the ultimate quality calculation. The proposed metric in [47] is used for the quality evaluation of various asymmetrically compressed stereoscopic 3D videos. It is observed that the results obtained from the proposed 2D-to-3D metric are more successful than the results obtained from the direct averaging method. The metric proposed in [48] uses two important phenomena (i.e., binocular suppression and recurrent excitation) to model the HVS better and improve depth perception. The FR 3D-video-quality metric proposed in [49] is based on measuring the directional dependency between the motion and depth sub-band coefficients of stereoscopic 3D videos. The proposed metric in [50] evaluates the quality of 3D videos synthesized with DIBR from three aspects: the quality of unoccluded regions, quality of first-order similarity, and quality of second-order similarity using an energy-based sequence-mapping strategy. Another SSIM-based metric in [51] uses the perceptually significant features, contrast, and motion characteristics that have an impact on the HVS.

### 2.2. NR Metrics

In [52,53], an objective metric (3VQM) is proposed for Depth-Image-Based Rendering (DIBR)-based stereoscopic 3D videos. According to this metric, firstly, the ideal depth map is estimated, which is then used to derive three distortion measures (temporal outliers—TO, temporal inconsistencies—TI, and spatial outliers—SO) to objectify the visual discomfort in the stereoscopic videos. The combination of the three measures constitutes a vision-based quality measure for 3D DIBR-based videos. In the metric proposed in [54], the four factors (temporal variance, disparity variation in the intraframes, disparity variation in the interframes, and disparity distribution in the frame-boundary areas) that affect human perception and visual comfort are examined. In [55], motion and parallax information obtained from depth maps and their histograms are the main parameters of the proposed stereoscopic VQA metric. The results show good performance for video sequences that contain annoying effects for the human eye.

In [56], an NR stereoscopic VQA metric that considers the correlation between the packet loss and perceptual video quality in the network is proposed. The metric yields better results than existing objective metrics so that it can be used in real time when monitoring network statistics. The NR metric proposed in [57], which can be used in the quality measurement of 3D videos that are corrupted or degraded after transmission, uses disparity-index-based dissimilarity measurements and edge-detection-based perceptual-difference measurements. Experimental results demonstrate the effectiveness of the proposed metric. In [58], a stereoscopic VQA metric is proposed to quantify the perceived quality of transmitted and degraded stereoscopic videos. The extracted features are accumulated according to the binocular suppression that is performed by measuring dissimilarity based on the disparity index and perceptual-difference measurement based on edge detection. According to the results, considering the effect of binocular rivalry in a stereoscopic video-quality metric seems to be effective at reflecting the HVS sensitivity and increasing the overall quality. 

The proposed NR metric in [59], which examines the effect of the variable network conditions on the 3D-video quality, uses the frame rate, bit rate, and network-packet-loss rate. In [60], the proposed NR metric considers the motion vector lengths and depth information for the 3D-video-quality evaluation. In [61], an NR 3D objective VQA metric that estimates the 3D quality by taking into account the spatial distortions, excessive disparity, depth representation, and temporal information of the video is proposed. The metric is resolution- and frame-rate-independent. To estimate the amount of spatial distortion in the video, the proposed metric computes blockiness. In [62], an extended NR objective 3D VQA metric that can run in real time is proposed. For this purpose, the network-packet loss, video-transmission bit rate, and frame-rate parameters are used as the input parameters. 

In [63], a stereo VQA metric by modeling the binocular perception effect in multiviews, including the spatial domain, temporal domain, and the spatial–temporal domain, is proposed. In [5], a depth-perception quality metric is applied to a blind stereoscopic video-quality evaluator to obtain an NR stereoscopic video-quality metric. The proposed NR metric in [64] is based on modeling the joint statistical dependencies between the motion and depth sub-band coefficients. In the proposed metric in [65], the components in the spatial and frequency domains associated with the HVS are used for the 3D VQA. In [66], the proposed NR stereoscopic VQA metric utilizes the 3D saliency map of the sum map first and then uses the sparse representation to decompose the sum map of 3D saliency into coefficients and calculates the features based on sparse coefficients to obtain the effective expression of the videos’ message. 

The study in [67] introduces a 3D convolutional-neural-network-based SVQA framework that can model not only local spatiotemporal information but also global temporal information with cubic-difference video patches as the input. In [68], a blind NR 3D VQA metric, which is based on the HVS mechanism and natural video statistics of 3D-video characteristics, is proposed. In [69], a stereoscopic VQA metric based on motion perception is proposed. In [70], a comprehensive stereoscopic VQA metric based on the joint contribution of multiple-domain information and a new interframe cross about spatiotemporal information is proposed.

Apart from these studies, the study in [71] examines the added value of using stereo saliency prediction in FR and NR quality-evaluation cases. 

### 2.3. Evaluation of the State-of-the-Art Studies

As can be seen from the elucidations above, there are three important limitations regarding the QoE evaluation of 3D videos from the depth-perception perspective. One of them is that it is very difficult to measure the depth cues with current rational methods and scientific approaches in 3D videos. Only a limited number of factors can be considered from a large number of factors affecting the human 3D-video QoE, and these factors are evaluated only within the limits permitted by well-known scientific approaches. Another limitation is that the results obtained from objective 3D-video QoE metrics do not correspond exactly to the 3D-viewing perception of an end user. Therefore, it would not be wrong to state that the most important problem with objective 3D QoE evaluation metrics is the lack of a high correlation with the human depth-viewing perception. The last major problem relies on the fact that the researchers’ habit of designing their proposed metrics relies solely on the traditional FR, RR, or NR approaches.

Considering the handicaps elucidated above, the 2D + DM-formed 3D-video QoE evaluation metric proposed in this study is designed by using spatial-resolution-associated depth cues, which have the ability to directly affect the depth perception of the viewer (i.e., the blurriness and motion information measured on the 2D-texture videos and the retinal-image size and convergence measured on the DM sequences). Moreover, while developing the proposed metric with an innovative approach, the NR and the RR types are integrated together to make a hybrid metric. In light of these facts, it could be easily stated that a remarkable hybrid 3D-video QoE evaluation metric, which uses depth cues from two difficult sources and is obtained by getting rid of the routine FR, RR, and NR classification approach that the researchers are stuck in, is developed in the proposed study. 

## 3. Proposed Hybrid 3D-Video QoE Evaluation Method

In this paper, we propose a hybrid 3D-video QoE evaluation metric that utilizes depth cues associated with spatial resolution (i.e., blurriness and motion information extracted from the 2D-texture videos, retinal-image size, and convergence extracted from the depth maps). 

We have a salient reason for our focus on the depth cues associated with spatial resolution in this study. In 3D videos, the depth-perception satisfaction of a viewer is at the forefront. Therefore, the viewer unwittingly encounters many depth cues. Because of having a high depth-cue density, it is a rule of thumb in developing a 3D-video QoE evaluation metric to design it based on the QoE factors that increase the depth perception of the viewer. Since a significant amount of these are closely related to spatial resolution, it is appropriate to start with the spatial resolution.

Spatial resolution can be defined as the number of pixels used for displaying a certain area of a digital image that shows a plane defining a finite volume in unlimited space. In a digital image, the smaller the area a pixel occupies on an object, the more pixels are used to represent that object. Accordingly, as the number of pixels per area (i.e., the spatial resolution) in a digital image increases, it is possible to display more detail [72].

Considering a digital image with different spatial-resolution versions, objects are represented with a greater number of pixels in the higher-spatial-resolution version of this image. Therefore, the pixel-related losses in the objects are less and the lines that highlight the objects appear more. As the objects become apparent, it becomes easier to distinguish them from their background and other objects. Thus, the viewer’s depth perception increases. In contrast, in the lower-spatial-resolution version, objects are represented with fewer pixels due to the use of larger pixels. The increase in pixel-related losses in objects results in the loss of detail in the image and a decrease in the viewer’s depth perception [72].

On the other hand, depth cues in 2D color videos and associated DM sequences cause the viewer to perceive more or less depth depending on the spatial resolution. As a matter of fact, the HVS obtains better-quality stereoscopic vision by perceiving the monocular and binocular cues that create depth perception more and more comfortably in the version with high spatial resolution. On the contrary, in the version with low spatial resolution, the cues that create depth perception disappear or become unnoticeable enough to the viewer. In this case, it is not possible to obtain a superior-quality stereoscopic view [73].

For the reasons explained above, the spatial resolution of the 3D videos is an important player that directly affects a viewer’s depth-perception experience. Therefore, the development of a 3D-video QoE evaluation metric, considering the role of this player in obtaining stereoscopic vision in the HVS, draws the attention of this research study.

**Figure 1:**
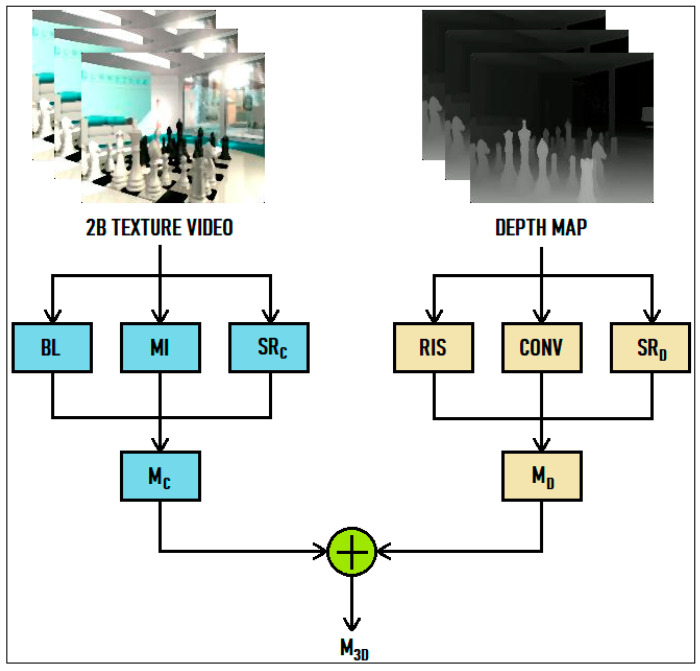
The framework of the proposed 3D-video QoE evolution metric.

The framework of the proposed 3D-video QoE evaluation metric is illustrated in Figure 1. As shown in the framework, we prefer using a 3D-video representation that is the product of the 2D + DM method. The 2D + DM method has become one of the most preferred 3D-video-creation techniques due to its support for coding, transmission, and compression technologies [74]. 

As can also be seen from Figure 1, due to the usage of 3D videos obtained with the 2D + DM method in this study, the proposed metric has two main elements, with one from the 2D-texture video ($M_{C}$) and the other from the DM ($M_{D}$). It is clear that these elements have their own effects on the viewer’s perception of depth, and each contributes separately to the artificial stereoscopic vision. A change in one of these elements directly causes the viewer’s depth perception to change. The reflection of this change in the artificial stereoscopic vision occurs independently of the other element. Therefore, there is an additive relationship between these elements, and this relationship can be illustrated in a metric created based on superposition theory. In light of these explanations, the proposed metric combines these two elements as follows:

(1) $$M_{3D}=M_{C}+M_{D}$$

where $M_{3D}$ is the proposed metric’s expansion.

The $M_{C}$ element provides the effects of two depth cues, blurriness and motion information, in the texture video and the spatial resolution of the 2D-texture video on the depth perception of the viewers. The $M_{D}$ component provides the contributions of the two monocular cues in the DM (i.e., the retinal-image size and convergence) and the spatial resolution to the depth perception of the viewers. The $M_{3D}$ value ranges from 0 to 15.

### 3.1. Proposed Models for the Depth Cues

As we state in Section 3, while we construct the proposed metric, we prefer using the 3D-video representation form, which is the product of the 2D + DM method. The 2D-texture videos are the main components of the 3D videos. While the main QoE factors that create depth perception in the viewer are depth cues hidden in the 2D-texture videos, the helping-component DM sequences have depth-information pixels corresponding to each pixel in the associated 2D-texture video. The quality of the 3D-video viewing experience of the viewers increases significantly with the effective use of these QoE factors or bringing these factors into the foreground. Therefore, it is indisputable that the QoE of the 3D videos that succeed in showing more realistic scenes to the viewer because of being equipped with depth cues is high.

The 2D + DM-formed 3D videos are tailor-made for measuring the depth cues. They allow for measuring the depth cues in the 2D-texture videos and DM sequences separately and provide the possibility to measure depth cues from two separate sources.

### 3.2. Blurriness

The blur defect, which directly affects the video quality of digital images, manifests itself as the reduction in high-frequency components containing edge information in the image. Accordingly, in digital images, the values of the neighbor pixels in the blurred parts of the images converge. 

The ambiguity that occurs especially in the edge information of the objects causes the shapes of the objects to not be understood by the viewer or the objects to be indistinguishable from each other or the background. This situation dramatically reduces the perception of depth of the viewer. Therefore, blurriness is an unacceptable flaw in 3D videos that can be associated with the spatial resolution of these videos.

In this study, to scale the blurriness, the total standard deviation of the 2D-texture videos is normalized by the spatial resolution and frame rate as follows:

(2) $$B=\sum_{i=1}^{f}\sqrt{\frac{1}{N}\sum_{j=1}^{N}{\left(x_{j}-\bar{x}\right)}^{2}\;}\frac{F}{S}$$

where B is the blurriness, i is the frame number, f is the total number of frames, j is the pixel number, N is the total number of pixels, $x_{j}$ is the pixel value, $\bar{x}$ is the mean of the pixel values in the frame, F is the frame rate, and S is the spatial resolution. Table 1 presents the blurriness measurements of 2D-texture videos calculated by using Equation (2). According to Table 1, the measurements show that the amount of blurriness in versions of a selected 2D-texture video (e.g., Breakdance) with any specific spatial resolution (e.g., SD) and with a gradually increasing compression ratio from QP = 25 to QP = 45 is close. It is also seen that the amount of blurriness in the versions of a selected 2D-texture video (e.g., Ballet) encoded with any specific compression ratio (e.g., QP = 25) and whose spatial resolution changes gradually from SD to QCIF fluctuates. These observations clearly state the correlation between blurriness and spatial resolution.

### 3.3. Motion Information

One of the most remarkable parameters affecting the depth perception of a viewer is the motion information of a 3D video. The motion information is a parameter that depends on the motion density of video frames.

The motion density in a frame is directly proportional to the spatial resolution of the frame. This is because the higher the spatial resolution of the frame, the higher the motion density of the frame.

Optical-flow vectors are used to measure the motion density of the frames. In the calculation of optical-flow vectors, dense or sparse optical-flow algorithms are used. The dense optical flow is based on the global calculation of the amount of displacement of each pixel in an image sequence that occurs between the current frame and the previous frame. Therefore, every pixel that is displaced and not displaced is included in the calculation. The sparse optical flow, on the other hand, is based on the local calculation of the displacement of only displaced pixels in an image sequence between the current frame and the previous frame.

In this study, we use an optical-flow vector calculated by using the Horn and Schunck method, which is a dense optical-flow algorithm, to measure the motion information.

The motion information is calculated by normalizing the average of the total motion density in a video sequence as follows [75]:

(3) $$M=\frac{\sum_{i=1}^{f}\Pi (i)}{f}\times \frac{F}{S}$$

where M is the motion information, i is the number of frames, f is the total number of frames, Π(i) is the motion density of the *i*-th video frame, F is the frame rate, and S is the spatial resolution. Π(i) is calculated according to the following equation [75]:

(4) $$\Pi \left(i\right)=\sum_{d=1}^{n}\left|V_{d}(x_{i},y_{i})\right|$$

where d is a feature point in the frame, n is the number of feature points in the frame, and $V_{d}(x_{i},\;y_{i})$ is the motion vector of the *i*-th frame at feature point d. Table 2 shows the motion-information measurements of 2D-texture videos computed by using Equation (3). In Table 2, it is noticeable that as the compression ratio gradually increases (from QP = 25 to QP = 45) in the SD, CIF, or QCIF spatial-resolution forms of each 3D video, the motion amount gradually decreases. A strong relationship between the motion information and compression ratio can be observed clearly. In addition, as the spatial resolution gradually changes (from QCIF to SD) at any QP value, the motion amount gradually increases. As can be seen, there is another strong relationship between the motion information and spatial resolution.

### 3.4. Retinal-Image Size

**Figure 2:**
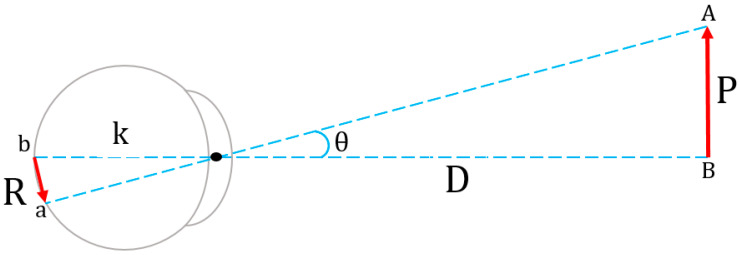
The geometry of Emmert’s law.

According to Emmert’s law [76], the distance between an object and its viewer can be calculated by using the actual size of the object and the size of its image on the viewer’s retina (see Figure 2).

**Figure 3:**
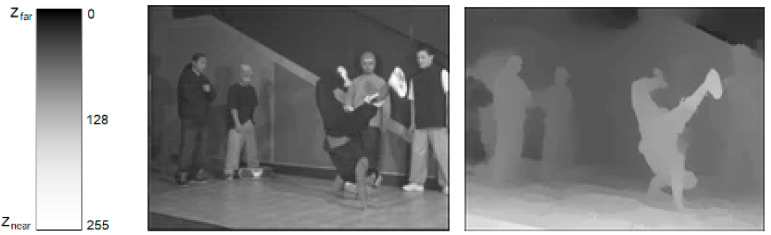
Change in depth values in Breakdance DM sequence.

The mathematical expression of this law is given by the following equation:

(5) P=R×D

where P is the size of the object, D is the distance of the object to the viewer’s eye, and R is the size of the image of the object formed on the retina. Since the P does not change, the R decreases when the D increases and vice versa. In other words, when the object moves away from the viewer and the depth increases, the retinal-image size of the object decreases, and the viewer perceives it as smaller. On the contrary, when the object moves nearer to the viewer and the depth decreases, the retinal-image size of the object increases, and the viewer perceives it as larger. This interesting phenomenon occurs as the change in the pixel values of the DM sequences of 3D videos occurs. While an object moves farther or nearer, the depth-pixel values change between 0 and 255 depending on the depth of the object, and the depth-pixel colors take gray tones. White corresponds to the nearest distance and black corresponds to the farthest distance (see Figure 3). 

In light of this information, it is proposed to use the change in the depth-pixel values in the DM to compute the retinal-image size in this study. This change can be calculated with the Mean Absolute Deviation (MAD) method for each DM frame as follows:

(6) $$R=\frac{\sum_{i=1}^{m}\sum_{j=1}^{n}\left|X_{i,j}-\bar{X}\right|}{m\times n}$$

where $X_{i,j}$ is the depth-pixel value at point (i,j); $\bar{X}$ is the average of the depth-pixel value of the frame of the DM sequence; and m and n are the width and height, respectively. Table 3 presents the retinal-image-size measurements of the DM sequences calculated considering Equation (6) and shows that the retinal-image-size measurements in the versions of a selected DM (e.g., Advertisement) with any specific spatial resolution (e.g., SD) and with a gradually increasing compression ratio from QP = 25 to QP = 45 gradually increase or tend to increase. This fluctuation means that there is no significant relationship between the retinal-image size and compression ratio. It is highly considered that this lack of relationship is caused by spatial and temporal distortions due to encoding, compressing, resizing, upsampling, downsampling, or other similar reasons in the DM sequences. Table 3 also shows that the retinal-image-size measurements in the versions of a selected DM (e.g., Butterfly) encoded with any specific compression ratio (e.g., QP = 25) and whose spatial resolution changes gradually from SD to QCIF gradually increase or tend to increase. This proves a strong relationship between the retinal-image size and spatial resolution.

### 3.5. Convergence

**Figure 4:**
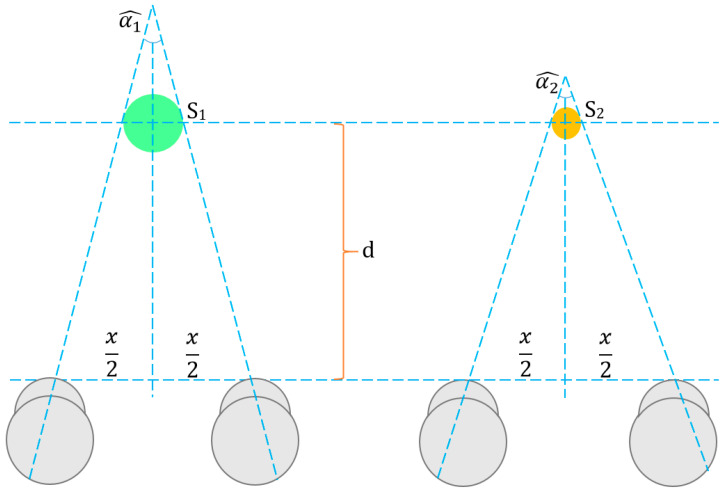
The geometry of convergence. The green and orange circles represent two objects having different sizes. The grey circles represent left and right eyes.

The position of objects affects the viewing angle of the eyes. Convergence is seeing an object that is moving closer to the viewer’s eyes with a greater angle. Therefore, convergence is a factor that directly increases the depth perception of the viewer. As seen in Figure 4, the viewing angle for an object positioned at d distance from the viewer is calculated as follows:

(7) $${\alpha =2\tan}^{-1}(\frac{x}{2d})$$

where α is the viewing angle and x is the distance between two human eyes. In the literature, the x distance between two human eyes is adopted as 65 mm [77].

The viewing angles differ for objects located at the same distance from the eye but with different volumes and surface areas. In Figure 4, two objects with different surface areas ($S_{1}$ > $S_{2}$) are positioned at the same distance d from the viewer. Accordingly, the viewing angle for the object with a larger surface area ($\alpha_{1}$) will be smaller than the viewing angle for the object with a smaller surface area ($\alpha_{2}$). This is similar for DM sequences with different spatial resolutions.

According to the geometric analysis in Figure 4, if the viewers watch SD-, CIF-, and QCIF-sized DM sequences of a 2D-texture video, they perceive that the distance of the objects does not change, but the objects in the DM sequences are reduced in size and settle in farther locations. This means that DM sequences with a lower spatial resolution are viewed with a larger viewing angle.

In order to obtain convergence in this study, the viewing angles are calculated by using Equation (7) for each frame of the DM sequences, and the total viewing angle is normalized as follows:

(8) $$C=\frac{\sum_{i=1}^{f}\alpha_{i}}{f\times S}$$

where C is the convergence, i is the number of frames, f is the total number of frames, S is the spatial resolution, and α is the angle of convergence. Table 4 presents the convergence measurements of DM sequences computed considering Equation (8) and shows that the convergence measurements in the versions of a selected DM (e.g., Interview) with any specific spatial resolution (e.g., SD) and with a gradually decreasing compression ratio from QP = 25 to QP = 45 fluctuate. Similar to the retinal-image-size clause, this fluctuation also means that there is no significant relationship between convergence and the compression ratio because of the reasons explained before. Table 4 also shows that the convergence measurements in the versions of a selected DM sequence (e.g., Windmill) encoded with any specific compression ratio (e.g., QP = 25) and whose spatial resolution changes gradually from SD to QCIF gradually increase or tend to increase. So, a strong relationship between convergence and the spatial resolution can be observed.

### 3.6. Subjective Tests

Subjective test results, conducted within the framework of standards adopted by major standard bodies, are in fact derived directly from the human vision system. Thus, it becomes possible to consider the relative effect of the depth cues on the viewers by using the subjective test results represented by the MOSs. In this study, subjective tests are conducted to construct a relationship between the MOS values and the proposed metric. After the tests, the 95% confidence intervals [78] are also computed together with the MOS values. 

The subjective tests were carried out independently of the metric design and by using 10 different 2D + DM-formed 3D videos (Breakdance, Ballet, Windmill, Newspaper, Interview, Advertisement, Butterfly, Chess, Farm, and Football) in different spatial resolutions (i.e., SD, CIF, and QCIF) encoded with 25, 30, 35, 40, and 45 Quantization Parameters (QPs). An autostereoscopic display of 23′ is utilized to present 2D + DM-form-based 3D videos during the experiments. 

Before the subjective tests, the participants are sufficiently informed about the features of the test and scoring. The scores given by the observers range from one to five. A five indicates that perception is at the highest level, and a one indicates that it is at the lowest level. The observers are not informed about the order, coding parameters, and features of the test videos. 

The observers participating in the tests do not have expertise in 3D videos. The observers participated in the test sitting 3 m away from the autostereoscopic screen. The tests are always carried out in the same test environment. To create the 3D videos, the same sized and encoded DM sequences and 2D-texture videos are used.

During the tests, the Single Stimulus Continuous Quality Evaluation (SSCQE) method is used for quality evaluation. The observers only evaluate the quality and depth perception of the encoded 3D video and the overall 3D-video quality separately without taking a 3D video as a reference. While making this evaluation, the observers benefited from their previous experiences. Inconsistent scores were obtained in all test results based on the ITU-R BT.500-13 standard [78]. Thus, the results of 2 of the 23 observers who participated in the test are determined to be inconsistent. The test results of the remaining 21 observers are used to calculate the MOS values.

## 4. Modeling of $M_{C}$ and $M_{D}$

### 4.1. Modeling of $M_{C}$

As stated above, the $M_{C}$ element, which represents the 2D-texture video QoE evolution component, combines two depth cues, namely the blurriness and motion information existing in a 2D (i.e., texture) video and the spatial resolution of the 2D video. In order to form a model for this element, the results of the subjective tests are integrated with the $M_{C}$ element to obtain a more-efficient 3D-video-quality metric. During this integration process, the best correlation between the subjective test results and the $M_{C}$ element is taken to determine the mathematical equation of $M_{C}$. The Pearson correlation method is used for this correlation calculation. The common feature of the depth cues is that they change when the spatial resolution changes. Therefore, a multiplicative relationship between the depth cues and the spatial resolution is considered to be the best reflection of the viewers’ depth perception considered in the proposed model. With this approach, the mathematical equation of $M_{C}$ is determined as follows:

(9) $$M_{C}=k_{C}\times B\times M\times S_{C}$$

where B and M are the blurriness and motion-information depth cues, respectively, and $S_{C}$ is the spatial resolution of the 2D-texture video. In addition, the $k_{C}$ constant coefficient in Equation (9) is selected as $10^{-4}$ for all 2D videos in order to keep the $M_{3D}$ values within the specified interval.

### 4.2. Modeling of $M_{D}$

As discussed above, the $M_{D}$ element, which states the DM-quality-evolution element, provides the contributions of the two monocular cues in the DM (i.e., the retinal-image size and convergence) and the spatial resolution to the depth perception of a viewer. To be able to construct a model for this element, similar to the process conducted for the $M_{C}$ element, the results of the subjective tests are integrated with the $M_{D}$ element that is adopted as the product of the two monocular cues and the spatial resolution of the DM sequences so as to make more contributions to the proposed metric. Similar to the $M_{C}$ model, the common feature of the monocular cues is that they vary when the spatial resolution varies, and a multiplicative relationship between the monocular cues and the spatial resolution is a useful assumption to reflect the viewers’ depth perception for the proposed model.

In this sense, the $M_{D}$ element’s mathematical model is formulated as follows:

(10) $$M_{D}=k_{D}\times R\times C\times S_{D}$$

where R and C are the retinal-image size and convergence monocular-depth cues, respectively, and $S_{D}$ is the spatial resolution of the DM. Also, the $k_{D}$ constant coefficient in Equation (10) is selected as $2.5\times 10^{8}$ for all DMs in order to keep the $M_{3D}$ values within the specified interval.

## 5. Results and Discussions

In this study, the last 150 frames of ten different 2D + DM-formed 3D videos (Breakdance, Ballet, Windmill, Newspaper, Interview, Advertisement, Butterfly, Chess, Farm, and Football) with different spatial resolutions (i.e., SD, CIF, and QCIF) and encoded with 25, 30, 35, 40, and 45 QPs are used to derive results from the proposed metric. The publicly available original versions of these videos were provided by the I-Lab, Center for Vision, Speech, and Signal Processing at the University of Surrey, UK, for research purposes. In order to evaluate the performance of the proposed metric, the MOS values and the quality-evaluation results of widely used 2D-video quality-evaluation metrics, namely the VQM, Peak Signal-to-Noise Ratio (PSNR), and structural-similarity metric (SSIM), are also calculated by using the same 3D videos. All video-quality measurements are set at a precision of four digits after the decimal point. 

Table 5, Table 6, Table 7, Table 8, Table 9, Table 10, Table 11, Table 12, Table 13 and Table 14 show the quality measurements of the videos used in terms of the MOS, VQM, PSNR, and SSIM results. The confidence-interval values for the MOS results are also presented in the tables. According to the MOS, VQM, PSNR, and SSIM results, it can be clearly observed that as the compression ratio gradually increases (from QP = 25 to QP = 45) in the SD, CIF, or QCIF spatial-resolution forms of each 3D video, the 3D-video QoE by the viewer decreases. This clearly shows the effects of the video spatial resolution and video compression ratio on the 3D-video QoE. The results obtained from the proposed metric bear a resemblance to the MOS results as well as the VQM, PSNR, and SSIM techniques. As can also be observed in the tables, the highest quality measurements calculated by the objective VQM, PSNR, and SSIM methods are obtained from the lowest compression ratio (QP = 25) versions of the SD, CIF, and QCIF spatial-resolution videos. As the compression ratio increases gradually, it is observed that the video quality decreases slightly at each compression level compared to the previous compression level. A similar situation is also observed in the gradual decrease in the MOS measurements obtained from subjective tests. From this point on, we will discuss the $M_{3D}$ measurements of the proposed metric.

Table 5 shows the quality measurements of the video “Breakdance”. The $M_{3D}$ measurement values obtained from the proposed metric are similar to both the objective video-quality measurements and subjective MOS measurements. In other words, as with other video-quality-measurement methods, the highest quality measurements in the proposed metric are obtained from the lowest compression ratio (QP = 25) version of the SD, CIF, and QCIF spatial-resolution videos. As the compression ratio gradually increases, the $M_{3D}$ measurement decreases. The same situation is observed for the video “Interview” in Table 7. 

Table 6 gives the quality measurements of the video “Ballet”. The $M_{3D}$ measurements obtained from the proposed metric generally show similarity to both the objective video-quality measurements and subjective MOS values. Only the $M_{3D}$ measurements for the QP = 30 and QP = 35 compression ratios at the CIF spatial resolution are equal. Here, the $M_{3D}$ measurement value for the QP = 35 compression ratio is expected to be low, but not lower than the $M_{3D}$ measurement value for the QP = 40 compression ratio, or the $M_{3D}$ measurement value for the QP = 30 compression ratio is expected to be high, but not higher than the $M_{3D}$ measurement value for the QP = 25 compression ratio. These expectations of the $M_{3D}$ measurements are true for all of the QP-related results, and this equality arises due to the fact that there are no huge deviations in the $M_{3D}$ measurement values for both compression ratios QP = 30 and QP = 35.

In Table 8, the quality measurements for the video “Newspaper” are given. The $M_{3D}$ measurements taken at the SD spatial resolution have a similar variation to other objective video-quality measurements and especially the MOS values. But the $M_{3D}$ measurements taken at the CIF spatial resolution for QP = 25, QP = 30, and QP = 35 are equal. Also, some deviations are observed in the $M_{3D}$ and SSIM measurements at the QCIF spatial resolution. These equalities in the CIF spatial resolution and deviations in the QCIF spatial resolution result from compression and downsampling processes for this video. However, these results look insignificant considering the number precision.

Table 9 demonstrates the quality measurements for the video “Windmill”. According to Table 9, only the $M_{3D}$ measurements taken at the SD and CIF spatial resolution for the QP = 25 and QP = 30 compression ratios show insignificant deviations that are not possible to be perceived by the HVS. Also, some insignificant deviations are observed at the QCIF spatial resolution.

Table 10, which gives the quality measurements of the video “Advertisement”, shows that the $M_{3D}$ measurements are not compatible with other objective video-quality measurements and the MOS values. The $M_{3D}$ measurements have huge deviations at all spatial resolutions for all compression ratios. But the video “Advertisement” is a CGI-based video, so the deviations most likely arise from the rendering method. The NSI-based video-quality-evolution metrics do not give accurate results in the quality measurements of the CGI-based videos.

The quality measurements of the video “Butterfly” are given in Table 11. According to this table, only the $M_{3D}$ measurements taken at an SD spatial resolution for the QP = 25 and QP = 30 compression ratios show deviation. This issue is most likely caused by errors in the compression process. The rest of the $M_{3D}$ measurements are aligned with the other objective quality measurements and the MOS values.

The measurements of the video “Chess” in Table 12 show that only the $M_{3D}$ measurements taken at the QCIF spatial resolution show similar variations to other objective video-quality measurements and the MOS values. But, there are significant deviations in the SD and QCIF spatial resolutions for all the compression ratios. These deviations are most likely caused by the rendering method, which makes “Chess” a CGI-based video. And, the quality of a CGI-based video should be measured by using a CGI-based video-quality-evaluation metric.

Table 13 gives the quality measurements of the video “Farm”. This table shows that only $M_{3D}$ measurements taken at the SD spatial resolution are aligned with the other objective video-quality measurements and the MOS values. Although there are bias-like deviations at the CIF and QCIF spatial resolutions, these deviations are too insignificant to be perceived by the HVS. On the other hand, the VQM, PSNR, and SSIM measurements have deviations at all spatial resolutions and for all compression ratios because of the errors in the encoding, compressing, and resizing processes.

Lastly, it is observed in Table 14 showing the measurements of the video “Football” that the $M_{3D}$ measurements taken at the SD spatial resolution for the QP = 25 or QP = 30 compression ratios show deviations. Also, although there is another deviation at the CIF and QCIF spatial resolutions, they are very small and thus cannot be perceived by the HVS.

**Figure 5:**
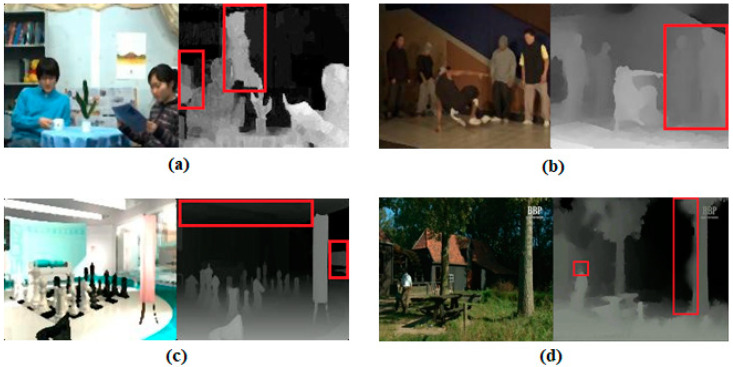
Some artifacts in the DMs of (**a**) Newspaper, (**b**) Breakdance, (**c**) Chess, and (**d**) Farm. The red rectangles/squares highlight some remarkable artifacts on the DM sequences.

As a general assessment according to the $M_{3D}$ measurements, the quality estimates of the proposed metric show significant similarities with the VQM, PSNR, and SSIM measurements and especially the MOS values. Approximately 80% of the results obtained from the proposed metric vary in accordance with the MOS, VQM, PSNR, and SSIM variances. The majority of the remaining 20% show insignificant variances that cannot be noticed by the HVS. It is considered that these cases are caused by spatial and temporal distortions due to encoding, compressing, resizing, upsampling, downsampling, pixel losses, or other similar reasons in the 2D-texture videos and DM sequences of the 3D videos used. Particularly, the effects of the change in the compression ratio on DM sequences are remarkable. In addition, the artifacts observed in some DM sequences led to inaccurate calculations of the depth cues and had disruptive effects on the $M_{3D}$ measurements (see Figure 5).

Moreover, the 3D-video QoE evaluation-performance efficiency of the $M_{3D}$ over the VQM, PSNR, and SSIM metrics can be observed from the correlation coefficient (CC) results calculated by using the MOS results. The CC results calculated by using the Pearson method and showing the relationship between the $M_{3D}$ quality estimations and the MOS values are given in Table 15. The average CC results of the $M_{3D}$ and the MOS are computed as 0.775 for all the 3D videos, QPs, and spatial resolutions. However, the CC results of the $M_{3D}$ and the VQM, PSNR, and SSIM metrics are computed as 0.784, 0.772, and 0.838, respectively. From this point on, we will take a deeper look at Table 15.

For the videos “Breakdance”, “Ballet”, “Interview”, “Football”, and “Butterfly”, the $M_{3D}$ measurements have high correlation coefficients with the objective VQM, PSNR, and SSIM metrics and subjective MOS measurements. This means that there are strong linear relationships between the $M_{3D}$ measurements and the other video-quality measurements used. 

The lowest correlation coefficients between the $M_{3D}$ measurements and the other video-quality measurements are observed in the video “Advertisement”. The correlation coefficients of the video “Advertisement” are generally below the value 0.3 so that there are weak linear relationships between the $M_{3D}$ measurements and other video-quality measurements. This also means that an increase in any video-quality measurement does not mean a higher $M_{3D}$ measurement and vice versa. 

For the videos “Farm” and “Chess”, half of the CC results are between 0.3 and 0.7, and the remaining half are above 0.7. As the CC results between 0.3 and 0.7 (half) indicate moderate linear relationships between the $M_{3D}$ measurements and the VQM, PSNR, SSIM, and MOS measurements, the CC results above 0.7 indicate strong linear relationships between the $M_{3D}$ measurements and the VQM, PSNR, SSIM, and MOS measurements.

For the videos “Windmill” and “Newspaper”, the CC results of the QCIF versions are generally below 0.3 so that there are weak linear relationships between the $M_{3D}$ measurements and the VQM, PSNR, SSIM, and MOS measurements. As mentioned above, this situation arises from the negative reflections on the $M_{3D}$ of the errors that occur in processes such as encoding, resizing, and downsampling. The CIF and SD versions have high CC results, which mean strong linear relationships between the $M_{3D}$ measurements and the VQM, PSNR, SSIM, and MOS measurements.

In light of the CC results in Table 15 and the explanations above, it is understood that there is a useful correlation between $M_{3D}$ quality estimations and the measurements of the MOS, VQM, PSNR, and SSIM; also, this correlation is worth considering when developing a new hybrid 3D-video-quality metric based on spatial resolution and depth cues.

## 6. Conclusions and Future Works

Researchers use subjective tests in general to evaluate the quality of 3D videos. However, subjective tests have significant disadvantages such as a high cost, being time consuming, and its unsuitability for real-time applications. For this reason, there is a great need for an objective and hybrid 3D-video QoE evaluation metric that is highly correlated with the HVS and has excellent alignment with the MOS. Therefore, for such a metric to be developed, it is a must to consider the effects of depth cues and spatial resolution, which directly affect the viewer’s depth perception. 

In this study, a hybrid 3D-video QoE evaluation metric was developed that employs the effects of spatial-resolution-associated blurriness, motion information, retinal-image size, convergence, and parameters on the depth perception of the viewers to be used in the quality evaluation of 3D videos obtained by using the 2D + DM method, which may be a preferred method by the researchers. Blurriness and motion information were derived from the 2D color-texture video while the retinal-image size and convergence are derived from the DM. Also, spatial resolution is derived from both the color-texture video and the DM.

This study emphasizes the critical role of the depth cues associated with spatial resolution in designing an effective 3D-video QoE metric. The results show that the proposed hybrid metric is quite successful and can be utilized to predict the 3D-video QoE. Obtaining successful results from the proposed metric proves that it is an appropriate approach to use depth cues and spatial resolution together as input parameters while developing a 3D-video QoE evaluation metric. Especially, a high correlation with the HVS also proves the validity of the proposed metric’s estimations. The proposed metric will allow researchers to avoid the high cost of subjective tests and save time. Also, it is feasible to use the proposed metric in real-time applications as it is a hybrid metric. For these reasons, it will accelerate the studies on 3D-video technologies and encourage future studies.

It has to be noted that the predicted MOS values are eligible to be enhanced. In future work, it is possible to fine tune the formulas by optimizing the coefficients, developing different models for measuring the depth cues, changing existing depth cues with other depth cues, and/or adding extra depth-cue elements to the proposed metric to further improve the results.

## Figures and Tables

**Table 1:** Blurriness measurements per QP and spatial resolution for the 2D-video sequences.

2D Video	Video Size	Quantization Parameter (QP)				
		25	30	35	40	45
Breakdance	Original	0.260	0.259	0.258	0.256	0.236
	SD	0.258	0.258	0.257	0.255	0.253
	CIF	0.258	0.257	0.256	0.255	0.253
	QCIF	0.256	0.255	0.255	0.254	0.251
Butterfly	Original	0.092	0.092	0.092	0.091	0.090
	SD	0.089	0.089	0.089	0.088	0.088
	CIF	0.086	0.086	0.086	0.086	0.086
	QCIF	0.083	0.083	0.083	0.083	0.083
Windmill	Original	0.200	0.200	0.199	0.199	0.197
	SD	0.198	0.198	0.197	0.197	0.195
	CIF	0.196	0.196	0.196	0.195	0.194
	QCIF	0.192	0.192	0.192	0.192	0.191
Chess	Original	0.335	0.335	0.335	0.335	0.335
	SD	0.365	0.365	0.365	0.365	0.365
	CIF	0.379	0.379	0.379	0.379	0.379
	QCIF	0.378	0.378	0.378	0.378	0.379
Interview	Original	0.199	0.199	0.199	0.198	0.197
	SD	0.197	0.197	0.197	0.196	0.195
	CIF	0.190	0.190	0.190	0.190	0.189
	QCIF	0.183	0.183	0.184	0.183	0.183
Advertisement	Original	0.282	0.281	0.281	0.281	0.280
	SD	0.289	0.288	0.288	0.288	0.287
	CIF	0.288	0.288	0.288	0.287	0.286
	QCIF	0.286	0.286	0.285	0.285	0.285
Farm	Original	0.298	0.298	0.298	0.298	0.298
	SD	0.297	0.297	0.297	0.297	0.297
	CIF	0.296	0.296	0.296	0.296	0.296
	QCIF	0.293	0.293	0.294	0.293	0.293
Football	Original	0.206	0.207	0.206	0.206	0.204
	SD	0.205	0.205	0.205	0.204	0.203
	CIF	0.202	0.202	0.202	0.202	0.201
	QCIF	0.197	0.197	0.198	0.198	0.197
Newspaper	Original	0.278	0.278	0.278	0.278	0.278
	SD	0.356	0.356	0.356	0.365	0.356
	CIF	0.363	0.363	0.363	0.362	0.362
	QCIF	0.362	0.362	0.362	0.362	0.361
Ballet	Original	0.641	0.640	0.638	0.635	0.628
	SD	0.655	0.653	0.650	0.644	0.631
	CIF	0.656	0.654	0.650	0.645	0.632
	QCIF	0.657	0.654	0.651	0.645	0.632

**Table 2:** Motion-information measurements per QP and spatial resolution for the 2D-video sequences.

2D Video	Video Size	Quantization Parameter (QP)				
		25	30	35	40	45
Breakdance	Original	0.311	0.333	0.309	0.282	0.224
	SD	0.326	0.326	0.297	0.270	0.210
	CIF	0.265	0.260	0.240	0.222	0.169
	QCIF	0.216	0.214	0.203	0.187	0.143
Butterfly	Original	1.610	1.627	1.596	1.520	1.426
	SD	1.673	1.687	1.656	1.581	1.478
	CIF	1.146	1.144	1.114	1.081	1.027
	QCIF	0.791	0.783	0.764	0.737	0.701
Windmill	Original	0.153	0.152	0.145	0.137	0.110
	SD	0.130	0.130	0.125	0.119	0.101
	CIF	0.067	0.067	0.067	0.067	0.061
	QCIF	0.035	0.036	0.036	0.036	0.033
Chess	Original	0.280	0.281	0.279	0.276	0.301
	SD	0.254	0.254	0.251	0.252	0.270
	CIF	0.168	0.170	0.170	0.168	0.172
	QCIF	0.120	0.122	0.121	0.117	0.116
Interview	Original	0.114	0.109	0.101	0.090	0.078
	SD	0.119	0.114	0.106	0.095	0.082
	CIF	0.066	0.064	0.061	0.057	0.050
	QCIF	0.038	0.037	0.036	0.033	0.031
Advertisement	Original	0.292	0.300	0.304	0.298	0.283
	SD	0.308	0.314	0.321	0.314	0.307
	CIF	0.231	0.236	0.241	0.240	0.234
	QCIF	0.176	0.179	0.180	0.180	0.174
Farm	Original	1.033	1.043	1.026	0.970	0.878
	SD	1.142	1.141	1.119	1.053	0.956
	CIF	0.767	0.767	0.755	0.723	0.661
	QCIF	0.465	0.467	0.465	0.459	0.432
Football	Original	1.336	1.443	1.337	1.257	1.296
	SD	1.466	1.520	1.319	1.172	1.170
	CIF	1.271	1.271	1.043	0.856	0.812
	QCIF	0.636	0.640	0.566	0.468	0.440
Newspaper	Original	0.465	0.455	0.437	0.400	0.347
	SD	0.355	0.352	0.343	0.319	0.281
	CIF	0.192	0.192	0.191	0.185	0.169
	QCIF	0.102	0.102	0.103	0.102	0.099
Ballet	Original	0.247	0.265	0.255	0.221	0.203
	SD	0.248	0.239	0.232	0.210	0.190
	CIF	0.180	0.168	0.163	0.153	0.140
	QCIF	0.119	0.114	0.114	0.103	0.090

**Table 3:** Retinal-image-size measurements per QP and spatial resolution for the DM sequences.

DM Sequence	DM Size	Quantization Parameter (QP)				
		25	30	35	40	45
Breakdance	Original	3.291 × 10^−7^	3.290 × 10^−7^	3.279 × 10^−7^	3.244 × 10^−7^	3.246 × 10^−7^
	SD	6.364 × 10^−7^	6.361 × 10^−7^	6.343 × 10^−7^	6.278 × 10^−7^	6.282 × 10^−7^
	CIF	2.520 × 10^−6^	2.521 × 10^−6^	2.513 × 10^−6^	2.498 × 10^−6^	2.501 × 10^−6^
	QCIF	9.979 × 10^−6^	9.979 × 10^−6^	9.939 × 10^−6^	9.861 × 10^−6^	9.893 × 10^−6^
Butterfly	Original	6.835 × 10^−8^	6.857 × 10^−8^	6.952 × 10^−8^	7.076 × 10^−8^	7.245 × 10^−8^
	SD	9.518 × 10^−8^	9.536 × 10^−8^	9.600 × 10^−8^	9.802 × 10^−8^	1.005 × 10^−7^
	CIF	3.767 × 10^−7^	3.793 × 10^−7^	3.810 × 10^−7^	3.918 × 10^−7^	4.019 × 10^−7^
	QCIF	1.489 × 10^−6^	1.503 × 10^−6^	1.513 × 10^−6^	1.529 × 10^−6^	1.580 × 10^−6^
Windmill	Original	1.232 × 10^−7^	1.232 × 10^−7^	1.235 × 10^−7^	1.235 × 10^−7^	1.254 × 10^−7^
	SD	1.573 × 10^−7^	1.582 × 10^−7^	1.579 × 10^−7^	1.578 × 10^−7^	1.604 × 10^−7^
	CIF	6.272 × 10^−7^	6.312 × 10^−7^	6.294 × 10^−7^	6.297 × 10^−7^	6.404 × 10^−7^
	QCIF	2.495 × 10^−6^	2.509 × 10^−6^	2.502 × 10^−6^	2.509 × 10^−6^	2.550 × 10^−6^
Chess	Original	9.743 × 10^−8^	9.670 × 10^−8^	9.753 × 10^−8^	9.787 × 10^−8^	1.008 × 10^−7^
	SD	1.252 × 10^−7^	1.243 × 10^−7^	1.252 × 10^−7^	1.253 × 10^−7^	1.291 × 10^−7^
	CIF	5.027 × 10^−7^	4.996 × 10^−7^	5.040 × 10^−7^	5.025 × 10^−7^	5.164 × 10^−7^
	QCIF	2.030 × 10^−6^	2.009 × 10^−6^	2.032 × 10^−6^	2.022 × 10^−6^	2.065 × 10^−6^
Interview	Original	7.915 × 10^−8^	7.929 × 10^−8^	8.014 × 10^−8^	8.236 × 10^−8^	8.405 × 10^−8^
	SD	8.240 × 10^−8^	8.259 × 10^−8^	8.340 × 10^−8^	8.556 × 10^−8^	8.731 × 10^−8^
	CIF	3.287 × 10^−7^	3.296 × 10^−7^	3.333 × 10^−7^	3.425 × 10^−7^	3.491 × 10^−7^
	QCIF	1.309 × 10^−6^	1.320 × 10^−6^	1.337 × 10^−6^	1.369 × 10^−6^	1.389 × 10^−6^
Advertisement	Original	4.246 × 10^−8^	4.285 × 10^−8^	4.348 × 10^−8^	4.451 × 10^−8^	4.577 × 10^−8^
	SD	6.156 × 10^−8^	6.191 × 10^−8^	6.248 × 10^−8^	6.385 × 10^−8^	6.595 × 10^−8^
	CIF	2.470 × 10^−7^	2.474 × 10^−7^	2.492 × 10^−7^	2.567 × 10^−7^	2.632 × 10^−7^
	QCIF	1.011 × 10^−6^	1.023 × 10^−6^	1.034 × 10^−6^	1.056 × 10^−6^	1.084 × 10^−6^
Farm	Original	1.217 × 10^−7^	1.214 × 10^−7^	1.206 × 10^−7^	1.203 × 10^−7^	1.226 × 10^−7^
	SD	1.562 × 10^−7^	1.558 × 10^−7^	1.546 × 10^−7^	1.543 × 10^−7^	1.568 × 10^−7^
	CIF	6.233 × 10^−7^	6.216 × 10^−7^	6.167 × 10^−7^	6.156 × 10^−7^	6.254 × 10^−7^
	QCIF	2.472 × 10^−6^	2.467 × 10^−6^	2.443 × 10^−6^	2.446 × 10^−6^	2.483 × 10^−6^
Football	Original	1.214 × 10^−7^	1.224 × 10^−7^	1.223 × 10^−7^	1.231 × 10^−7^	1.241 × 10^−7^
	SD	1.551 × 10^−7^	1.563 × 10^−7^	1.563 × 10^−7^	1.573 × 10^−7^	1.586 × 10^−7^
	CIF	6.220 × 10^−7^	6.269 × 10^−7^	6.268 × 10^−7^	6.309 × 10^−7^	6.363 × 10^−7^
	QCIF	2.497 × 10^−6^	2.517 × 10^−6^	2.519 × 10^−6^	2.534 × 10^−6^	2.557 × 10^−6^
Newspaper	Original	1.646 × 10^−7^	1.659 × 10^−7^	1.670 × 10^−7^	1.692 × 10^−7^	1.718 × 10^−7^
	SD	3.258 × 10^−7^	3.268 × 10^−7^	3.278 × 10^−7^	3.311 × 10^−7^	3.354 × 10^−7^
	CIF	1.310 × 10^−6^	1.314 × 10^−6^	1.317 × 10^−6^	1.329 × 10^−6^	1.348 × 10^−6^
	QCIF	5.285 × 10^−6^	5.298 × 10^−6^	5.312 × 10^−6^	5.358 × 10^−6^	5.438 × 10^−6^
Ballet	Original	4.583 × 10^−7^	4.585 × 10^−7^	4.577 × 10^−7^	4.568 × 10^−7^	4.546 × 10^−7^
	SD	8.847 × 10^−7^	8.849 × 10^−7^	8.835 × 10^−7^	8.820 × 10^−7^	8.773 × 10^−7^
	CIF	3.559 × 10^−6^	3.560 × 10^−6^	3.555 × 10^−6^	3.547 × 10^−6^	3.535 × 10^−6^
	QCIF	1.412 × 10^−5^	1.413 × 10^−5^	1.411 × 10^−5^	1.410 × 10^−5^	1.407 × 10^−5^

**Table 4:** Convergence measurements per QP and spatial resolution for the DM sequences.

DM Sequence	DM Size	Quantization Parameter (QP)				
		25	30	35	40	45
Breakdance	Original	2.035 × 10^−10^	2.035 × 10^−10^	2.034 × 10^−10^	2.030 × 10^−10^	2.031 × 10^−10^
	SD	3.949 × 10^−10^	3.950 × 10^−10^	3.946 × 10^−10^	3.939 × 10^−10^	3.940 × 10^−10^
	CIF	1.582 × 10^−9^	1.582 × 10^−9^	1.581 × 10^−9^	1.577 × 10^−9^	1.578 × 10^−9^
	QCIF	6.362 × 10^−9^	6.362 × 10^−9^	6.350 × 10^−9^	6.329 × 10^−9^	6.335 × 10^−9^
Butterfly	Original	1.618 × 10^−10^	1.162 × 10^−10^	1.627 × 10^−10^	1.637 × 10^−10^	1.635 × 10^−10^
	SD	2.138 × 10^−10^	2.140 × 10^−10^	2.145 × 10^−10^	2.162 × 10^−10^	2.157 × 10^−10^
	CIF	8.559 × 10^−10^	8.577 × 10^−10^	8.599 × 10^−10^	8.672 × 10^−10^	8.648 × 10^−10^
	QCIF	3.420 × 10^−9^	3.433 × 10^−9^	3.442 × 10^−9^	3.452 × 10^−9^	3.448 × 10^−9^
Windmill	Original	2.795 × 10^−10^	2.799 × 10^−10^	2.781 × 10^−10^	2.772 × 10^−10^	2.790 × 10^−10^
	SD	3.578 × 10^−10^	3.583 × 10^−10^	3.559 × 10^−10^	3.548 × 10^−10^	3.573 × 10^−10^
	CIF	1.432 × 10^−9^	1.435 × 10^−9^	1.425 × 10^−9^	1.421 × 10^−9^	1.433 × 10^−9^
	QCIF	5.750 × 10^−9^	5.759 × 10^−9^	5.721 × 10^−9^	5.702 × 10^−9^	5.767 × 10^−9^
Chess	Original	3.151 × 10^−10^	3.114 × 10^−10^	3.104 × 10^−10^	3.047 × 10^−10^	3.003 × 10^−10^
	SD	4.041 × 10^−10^	3.994 × 10^−10^	3.977 × 10^−10^	3.900 × 10^−10^	3.844 × 10^−10^
	CIF	1.617 × 10^−9^	1.599 × 10^−9^	1.593 × 10^−9^	1.560 × 10^−9^	1.536 × 10^−9^
	QCIF	6.482 × 10^−9^	6.401 × 10^−9^	6.390 × 10^−9^	6.249 × 10^−9^	6.134 × 10^−9^
Interview	Original	1.972 × 10^−10^	1.975 × 10^−10^	1.981 × 10^−10^	1.989 × 10^−10^	1.998 × 10^−10^
	SD	2.028 × 10^−10^	2.031 × 10^−10^	2.034 × 10^−10^	2.045 × 10^−10^	2.054 × 10^−10^
	CIF	8.117 × 10^−10^	8.129 × 10^−10^	8.156 × 10^−10^	8.189 × 10^−10^	8.226 × 10^−10^
	QCIF	3.244 × 10^−9^	3.248 × 10^−9^	3.261 × 10^−9^	3.275 × 10^−9^	3.287 × 10^−9^
Advertisement	Original	1.178 × 10^−10^	1.183 × 10^−10^	1.193 × 10^−10^	1.207 × 10^−10^	1.233 × 10^−10^
	SD	1.524 × 10^−10^	1.530 × 10^−10^	1.542 × 10^−10^	1.560 × 10^−10^	1.594 × 10^−10^
	CIF	6.094 × 10^−10^	6.118 × 10^−10^	6.166 × 10^−10^	6.242 × 10^−10^	6.374 × 10^−10^
	QCIF	2.437 × 10^−9^	2.449 × 10^−9^	2.469 × 10^−9^	2.497 × 10^−9^	2.553 × 10^−9^
Farm	Original	2.182 × 10^−10^	2.181 × 10^−10^	2.178 × 10^−10^	2.185 × 10^−10^	2.200 × 10^−10^
	SD	2.799 × 10^−10^	2.800 × 10^−10^	2.795 × 10^−10^	2.804 × 10^−10^	2.823 × 10^−10^
	CIF	1.122 × 10^−9^	1.122 × 10^−9^	1.120 × 10^−9^	1.124 × 10^−9^	1.131 × 10^−9^
	QCIF	4.518 × 10^−9^	4.517 × 10^−9^	4.504 × 10^−9^	4.520 × 10^−9^	4.551 × 10^−9^
Football	Original	2.694 × 10^−10^	2.685 × 10^−10^	2.681 × 10^−10^	2.666 × 10^−10^	2.646 × 10^−10^
	SD	3.445 × 10^−10^	3.433 × 10^−10^	3.428 × 10^−10^	3.400 × 10^−10^	3.384 × 10^−10^
	CIF	1.378 × 10^−9^	1.373 × 10^−9^	1.371 × 10^−9^	1.364 × 10^−9^	1.353 × 10^−9^
	QCIF	5.514 × 10^−9^	5.494 × 10^−9^	5.485 × 10^−9^	5.458 × 10^−9^	5.415 × 10^−9^
Newspaper	Original	7.960 × 10^−11^	7.980 × 10^−11^	7.990 × 10^−11^	8.020 × 10^−11^	8.050 × 10^−11^
	SD	1.554 × 10^−10^	1.555 × 10^−10^	1.556 × 10^−10^	1.560 × 10^−10^	1.566 × 10^−10^
	CIF	6.223 × 10^−10^	6.225 × 10^−10^	6.225 × 10^−10^	6.240 × 10^−10^	6.261 × 10^−10^
	QCIF	2.493 × 10^−9^	2.492 × 10^−9^	2.493 × 10^−10^	2.497 × 10^−9^	2.505 × 10^−9^
Ballet	Original	2.313 × 10^−10^	2.316 × 10^−10^	2.315 × 10^−10^	2.313 × 10^−10^	2.316 × 10^−10^
	SD	4.493 × 10^−10^	4.498 × 10^−10^	4.496 × 10^−10^	4.492 × 10^−10^	4.496 × 10^−10^
	CIF	1.803 × 10^−9^	1.805 × 10^−9^	1.804 × 10^−9^	1.803 × 10^−9^	1.805 × 10^−9^
	QCIF	7.240 × 10^−9^	7.247 × 10^−9^	7.246 × 10^−9^	7.239 × 10^−9^	7.243 × 10^−9^

**Table 5:** Breakdance 3D-video QoE measurements.

Video	Quantization Parameter (QP)	Spatial Resolution (2D + DM)	M_3D_	VQM	PSNR	SSIM	MOS
Breakdance	25	SD	3.4401	4.8796	56.5618	0.9989	3.032 ± 0.32
	30		3.4332	4.8291	53.5868	0.9984	2.844 ± 0.29
	35		3.1140	4.7497	50.4148	0.9975	2.688 ± 0.35
	40		2.8213	4.6262	47.1143	0.9960	2.500 ± 0.33
	45		2.1744	4.4271	43.6735	0.9934	2.375 ± 0.28
	25	CIF	0.7920	4.9037	56.9623	0.9991	3.032 ± 0.34
	30		0.7777	4.8638	54.1026	0.9987	2.844 ± 0.35
	35		0.7248	4.8078	50.9981	0.9978	2.688 ± 0.31
	40		0.6745	4.7133	47.6829	0.9962	2.500 ± 0.27
	45		0.5342	4.5608	44.2103	0.9931	2.375 ± 0.32
	25	QCIF	0.5422	4.9125	56.8838	0.9993	3.032 ± 0.31
	30		0.5406	4.8809	54.2220	0.9989	2.844 ± 0.33
	35		0.5308	4.8346	51.3094	0.9983	2.688 ± 0.36
	40		0.5156	4.7579	48.1342	0.9969	2.500 ± 0.34
	45		0.4883	4.6352	44.6945	0.9942	2.375 ± 0.37

**Table 6:** Ballet 3D-video QoE measurements.

Video	Quantization Parameter (QP)	Spatial Resolution (2D + DM)	M_3D_	VQM	PSNR	SSIM	MOS
Ballet	25	SD	6.6267	4.8773	53.7605	0.9991	3.407 ± 0.27
	30		6.3759	4.8239	53.2095	0.9987	3.250 ± 0.29
	35		6.1400	4.7416	49.9250	0.9979	3.126 ± 0.32
	40		5.5372	4.6091	46.4618	0.9965	3.032 ± 0.35
	45		4.9082	4.4217	42.9127	0.9941	2.907 ± 0.36
	25	CIF	1.3605	4.9039	56.5066	0.9993	3.407 ± 0.42
	30		1.2735	4.8588	53.5567	0.9989	3.250 ± 0.39
	35		1.2735	4.7992	50.2766	0.9981	3.126 ± 0.37
	40		1.1640	4.7027	46.7613	0.9968	3.032 ± 0.33
	45		1.0611	4.5688	43.2264	0.9942	2.907 ± 0.35
	25	QCIF	0.8464	4.9150	56.5325	0.9995	3.407 ± 0.29
	30		0.8380	4.8773	53.6213	0.9992	3.250 ± 0.32
	35		0.8354	4.8303	50.5181	0.9987	3.126 ± 0.34
	40		0.8143	4.7481	46.9328	0.9976	3.032 ± 0.28
	45		0.7891	4.6356	43.4891	0.9956	2.907 ± 0.35

**Table 7:** Interview 3D-video QoE measurements.

Video	Quantization Parameter (QP)	Spatial Resolution (2D + DM)	M_3D_	VQM	PSNR	SSIM	MOS
Interview	25	SD	0.9474	4.2200	41.4998	0.9966	4.001 ± 0.27
	30		0.9075	4.1903	41.3180	0.9959	3.907 ± 0.33
	35		0.8448	4.1317	40.9247	0.9944	3.813 ± 0.31
	40		0.7592	4.0538	40.1647	0.9917	3.688 ± 0.29
	45		0.6530	3.8449	38.7946	0.9864	3.625 ± 0.32
	25	CIF	0.1345	4.3272	42.7706	0.9975	4.001 ± 0.28
	30		0.1303	4.3058	42.5561	0.9969	3.907 ± 0.26
	35		0.1253	4.2687	42.1289	0.9958	3.813 ± 0.31
	40		0.1164	4.2066	41.2602	0.9936	3.688 ± 0.29
	45		0.1037	4.0760	39.7609	0.9891	3.625 ± 0.33
	25	QCIF	0.0447	4.4151	42.3809	0.9973	4.001 ± 0.34
	30		0.0445	4.4017	42.2283	0.9970	3.907 ± 0.31
	35		0.0443	4.3779	41.9024	0.9962	3.813 ± 0.29
	40		0.0439	4.3343	41.2490	0.9948	3.688 ± 0.30
	45		0.0432	4.2470	40.0028	0.9915	3.625 ± 0.28

**Table 8:** Newspaper 3D-video QoE measurements.

Video	Quantization Parameter (QP)	Spatial Resolution (2D + DM)	M_3D_	VQM	PSNR	SSIM	MOS
Newspaper	25	SD	5.1342	4.0684	35.8210	0.9933	3.688 ± 0.41
	30		5.0837	4.0339	35.6992	0.9918	3.626 ± 0.40
	35		4.9536	3.9709	35.4685	0.9889	3.500 ± 0.38
	40		4.7325	3.8708	35.0251	0.9836	3.344 ± 0.36
	45		4.0618	3.7132	34.2498	0.9765	3.250 ± 0.34
	25	CIF	0.7252	4.0660	35.6577	0.9917	3.688 ± 0.33
	30		0.7252	4.0507	35.5944	0.9915	3.626 ± 0.29
	35		0.7252	4.0205	35.4349	0.9902	3.500 ± 0.31
	40		0.6999	3.9645	35.0834	0.9866	3.344 ± 0.32
	45		0.6422	3.8515	34.4194	0.9804	3.250 ± 0.30
	25	QCIF	0.1768	4.0558	35.2781	0.9892	3.688 ± 0.29
	30		0.1773	4.0504	35.2539	0.9894	3.626 ± 0.31
	35		0.1781	4.0343	35.1658	0.9892	3.500 ± 0.34
	40		0.1781	4.0066	34.9285	0.9879	3.344 ± 0.33
	45		0.1770	3.9303	34.9087	0.9843	3.250 ± 0.36

**Table 9:** Windmill 3D-video QoE measurements.

Video	Quantization Parameter (QP)	Spatial Resolution (2D + DM)	M_3D_	VQM	PSNR	SSIM	MOS
Windmill	25	SD	1.0434	4.4358	45.1454	0.9963	3.969 ± 0.26
	30		1.0445	4.3923	43.5693	0.9953	3.844 ± 0.23
	35		1.0045	4.3052	41.6618	0.9934	3.751 ± 0.28
	40		0.9569	4.1879	39.7736	0.9902	3.594 ± 0.31
	45		0.8033	3.9880	37.6751	0.9845	3.500 ± 0.29
	25	CIF	0.1566	4.4552	45.4161	0.9961	3.969 ± 0.30
	30		0.1570	4.4276	44.0346	0.9955	3.844 ± 0.32
	35		0.1553	4.3616	42.3003	0.9940	3.751 ± 0.34
	40		0.1546	4.2743	40.4856	0.9913	3.594 ± 0.36
	45		0.1433	4.1308	38.3891	0.9859	3.500 ± 0.31
	25	QCIF	0.1079	4.4626	45.2037	0.9955	3.969 ± 0.29
	30		0.1091	4.4407	43.9034	0.9950	3.844 ± 0.27
	35		0.1082	4.3906	42.3530	0.9940	3.751 ± 0.32
	40		0.1080	4.3181	40.7104	0.9921	3.594 ± 0.33
	45		0.1089	4.2043	38.8237	0.9879	3.500 ± 0.30

**Table 10:** Advertisement 3D-video QoE measurements.

Video	Quantization Parameter (QP)	Spatial Resolution (2D + DM)	M_3D_	VQM	PSNR	SSIM	MOS
Advertisement	25	SD	3.6042	4.8873	56.6045	0.9995	4.219 ± 0.42
	30		3.6778	4.8289	53.1858	0.9990	4.063 ± 0.39
	35		3.7451	4.7360	49.6790	0.9982	3.844 ± 0.33
	40		3.6653	4.5838	45.7916	0.9966	3.719 ± 0.35
	45		3.5679	4.3204	41.5741	0.9932	3.563 ± 0.38
	25	CIF	0.6777	4.9107	56.7655	0.9996	4.219 ± 0.36
	30		0.6909	4.8654	53.4021	0.9993	4.063 ± 0.33
	35		0.7072	4.7999	49.9854	0.9986	3.844 ± 0.31
	40		0.7017	4.6908	46.1555	0.9974	3.719 ± 0.29
	45		0.6829	4.4971	42.0431	0.9945	3.563 ± 0.27
	25	QCIF	0.1433	4.9176	56.5843	0.9997	4.219 ± 0.34
	30		0.1454	4.8774	53.2847	0.9995	4.063 ± 0.37
	35		0.1461	4.8215	50.0120	0.9990	3.844 ± 0.35
	40		0.1468	4.7272	46.2116	0.9981	3.719 ± 0.38
	45		0.1430	4.5706	42.2322	0.9962	3.563 ± 0.33

**Table 11:** Butterfly 3D-video QoE measurements.

Video	Quantization Parameter (QP)	Spatial Resolution (2D + DM)	M_3D_	VQM	PSNR	SSIM	MOS
Butterfly	25	SD	6.0256	4.8873	56.6045	0.9995	4.219 ± 0.35
	30		6.0710	4.8289	53.1858	0.9990	4.063 ± 0.38
	35		5.9588	4.7360	49.6790	0.9982	3.844 ± 0.32
	40		5.6684	4.5838	45.7916	0.9966	3.719 ± 0.34
	45		5.2647	4.3204	41.5741	0.9932	3.563 ± 0.37
	25	CIF	1.0064	4.9107	56.7655	0.9996	4.219 ± 0.40
	30		1.0032	4.8654	53.4021	0.9993	4.063 ± 0.42
	35		0.9786	4.7999	49.9854	0.9986	3.844 ± 0.39
	40		0.9498	4.6908	46.1555	0.9974	3.719 ± 0.43
	45		0.8998	4.4971	42.0431	0.9945	3.563 ± 0.37
	25	QCIF	0.1985	4.9176	56.5843	0.9997	4.219 ± 0.35
	30		0.1970	4.8774	53.2847	0.9995	4.063 ± 0.33
	35		0.1936	4.8215	50.0120	0.9990	3.844 ± 0.37
	40		0.1886	4.7272	46.2116	0.9981	3.719 ± 0.32
	45		0.1821	4.5706	42.2322	0.9962	3.563 ± 0.30

**Table 12:** Chess 3D-video QoE measurements.

Video	Quantization Parameter (QP)	Spatial Resolution (2D + DM)	M_3D_	VQM	PSNR	SSIM	MOS
Chess	25	SD	3.7715	4.8789	54.5879	0.9991	4.407 ± 0.33
	30		3.7714	4.8166	50.9710	0.9982	4.188 ± 0.30
	35		3.7181	4.7283	47.3275	0.9965	4.032 ± 0.29
	40		3.7373	4.5733	43.5673	0.9929	3.875 ± 0.32
	45		4.0090	4.3055	39.6801	0.9859	3.751 ± 0.35
	25	CIF	0.6665	4.9000	54.7785	0.9993	4.407 ± 0.36
	30		0.6731	4.8405	51.2166	0.9986	4.188 ± 0.38
	35		0.6718	4.7685	47.6791	0.9973	4.032 ± 0.41
	40		0.6663	4.6611	44.0473	0.9944	3.875 ± 0.37
	45		0.6809	4.4613	40.2214	0.9886	3.751 ± 0.39
	25	QCIF	0.1987	4.9089	54.6848	0.9996	4.407 ± 0.33
	30		0.1984	4.8524	51.1796	0.9990	4.188 ± 0.31
	35		0.1982	4.7830	47.6853	0.9980	4.032 ± 0.35
	40		0.1923	4.6834	44.1676	0.9959	3.875 ± 0.34
	45		0.1913	4.5261	40.4934	0.9915	3.751 ± 0.38

**Table 13:** Farm 3D-video QoE measurements.

Video	Quantization Parameter (QP)	Spatial Resolution (2D + DM)	M_3D_	VQM	PSNR	SSIM	MOS
Farm	25	SD	13.7420	4.8592	55.3304	0.9989	4.063 ± 0.31
	30		13.7356	4.3882	44.3243	0.9956	3.938 ± 0.34
	35		13.4822	4.6924	49.1877	0.9969	3.844 ± 0.36
	40		12.6759	4.5258	45.9308	0.9944	3.719 ± 0.29
	45		11.5130	4.2681	42.2888	0.9897	3.563 ± 0.35
	25	CIF	2.3161	4.8730	53.6109	0.9990	4.063 ± 0.32
	30		2.3176	4.4372	44.6049	0.9957	3.938 ± 0.29
	35		2.3176	4.7578	49.1674	0.9974	3.844 ± 0.35
	40		2.1857	4.6416	46.3153	0.9954	3.719 ± 0.31
	45		2.0014	4.4426	42.9054	0.9910	3.563 ± 0.28
	25	QCIF	0.4162	4.8496	51.0768	0.9988	4.063 ± 0.32
	30		0.4179	4.4530	44.4586	0.9955	3.938 ± 0.33
	35		0.4156	4.7571	47.6791	0.9976	3.844 ± 0.29
	40		0.4109	4.6676	45.5833	0.9958	3.719 ± 0.27
	45		0.3928	4.5059	42.8398	0.9920	3.563 ± 0.30

**Table 14:** Football 3D-video QoE measurements.

Video	Quantization Parameter (QP)	Spatial Resolution (2D + DM)	M_3D_	VQM	PSNR	SSIM	MOS
Football	25	SD	12.1641	4.8734	55.2868	0.9987	3.407 ± 0.23
	30		12.6200	4.8057	52.1508	0.9976	3.251 ± 0.26
	35		10.9462	4.7125	49.0173	0.9959	3.157 ± 0.28
	40		9.6997	4.5733	45.7945	0.9935	2.969 ± 0.22
	45		9.6318	4.3229	41.9282	0.9890	2.844 ± 0.25
	25	CIF	2.6262	4.9018	55.9488	0.9991	3.407 ± 0.27
	30		2.6288	4.8516	53.0047	0.9984	3.251 ± 0.25
	35		2.6288	4.7890	49.9781	0.9973	3.157 ± 0.22
	40		1.7745	4.6903	46.7740	0.9953	2.969 ± 0.24
	45		1.6785	4.5151	44.4051	0.9916	2.844 ± 0.28
	25	QCIF	0.4051	4.9066	55.8213	0.9993	3.407 ± 0.30
	30		0.4077	4.8604	53.0627	0.9988	3.251 ± 0.32
	35		0.3708	4.8119	50.2311	0.9980	3.157 ± 0.35
	40		0.3219	4.7342	47.1955	0.9965	2.969 ± 0.38
	45		0.3076	4.5929	43.4736	0.9935	2.844 ± 0.37

**Table 15:** Correlation between the M_3D_ measurements and the values of the MOS, VQM, PSNR, and SSIM.

3D Video	Spatial Resolution	Correlation between the M_3D_ and the MOS	Correlation between the M_3D_ and VQM	Correlation between the M_3D_ and PSNR	Correlation between the M_3D_ and SSIM
Breakdance	SD	0.924	0.994	0.953	0.996
	CIF	0.919	0.994	0.952	0.998
	QCIF	0.920	0.996	0.956	0.998
Ballet	SD	0.956	0.998	0.990	0.993
	CIF	0.956	0.984	0.971	0.970
	QCIF	0.925	0.992	0.957	0.995
Windmill	SD	0.887	0.979	0.916	0.987
	CIF	0.785	0.920	0.840	0.952
	QCIF	0.255	0.273	0.260	0.332
Newspaper	SD	0.907	0.982	0.991	0.978
	CIF	0.854	0.976	0.981	0.986
	QCIF	0.286	0.106	0.282	0.264
Interview	SD	0.977	0.978	0.985	0.983
	CIF	0.962	0.993	0.994	0.990
	QCIF	0.944	0.998	0.997	0.993
Advertisement	SD	0.134	0.450	0.239	0.513
	CIF	0.328	0.007	0.228	0.106
	QCIF	0.111	0.187	0.033	0.289
Butterfly	SD	0.880	0.984	0.922	0.989
	CIF	0.940	0.997	0.966	0.992
	QCIF	0.959	0.998	0.982	0.987
Chess	SD	0.525	0.783	0.601	0.826
	CIF	0.562	0.684	0.590	0.699
	QCIF	0.876	0.926	0.905	0.912
Farm	SD	0.939	0.675	0.646	0.936
	CIF	0.901	0.540	0.651	0.914
	QCIF	0.862	0.405	0.620	0.855
Football	SD	0.911	0.880	0.914	0.880
	CIF	0.906	0.902	0.882	0.903
	QCIF	0.959	0.939	0.959	0.925

## Data Availability

The publicly available videos were provided by the I-Lab, Center for Vision, Speech, and Signal Processing at the University of Surrey, UK, for research purposes.
